# Risk factors and short-term complications of high-grade intraventricular hemorrhages in preterm neonates in training hospitals of Alborz

**DOI:** 10.22037/ijcn.v15i1.20346

**Published:** 2021

**Authors:** Alireza JASHNI MOTLAGH, Azamolmolouk ELSAGH, Elham SEDIGHIPOOR, Mostafa QORBANI

**Affiliations:** 1 Neonatologist, Department of Neonatology, Alborz University of Medical Sciences, Karaj, Iran.; 2 MSN, Faculty of Nursing, Alborz University of Medical Sciences, Karaj, Iran.; 3 Department of Nursing, Faculty of Nursing & Midwifery, Tehran Azad University of Medical Sciences,Tehran, Iran.; 4 Department of Neonatal Intensive Care Unit, Mousavi Hospital, Zanjan University of Medical Sciences, Zanjan, Iran.; 5 Non-Communicable Disease Research Center, Alborz University of Medical Sciences, Karaj, Iran.

**Keywords:** Risk factor, Complications, Intraventricular Hemorrhage, Preterm

## Abstract

**Objectives:**

The aim of this study is to determine risk factors and short-term complications of high-grade intraventricular hemorrhages (IVHs) in preterm neonates. Other topics of investigation include the increase in complications of IVH with its severity and the effect of IVH risk factors on the severity of IVH.

**Materials & Methods:**

We conducted a retrospective case-control study of 436 consecutive preterm neonates with high-grade (3, 4) IVHs admitted in training hospitals of Alborz University in Karaj, Iran, from 2012 to 2017. The risk factors and short-term complications were assessed and analyzed in the subjects by SPSS 19.

**Results:**

Out of 10 000 eligible neonates, we identified 1203 premature infants with IVH. A total of 436 infants with IVH grades 3 and 4 were allocated to the case group. The control group consisted of 767 infants with IVH grades 1 and 2. This study revealed that the most common risk factors of IVH include lack of corticosteroid use in 67.2%, low Apgar score in 10%, and surfactant use in 5.7% of the patients. Ten percent (31 cases) had short-term complications (18 hydrocephalus and 13 death cases). Male gender (*P *= .006) and lower gestational age (*P *= .0001) contributed to higher grades of IVH.

**Conclusion:**

According to the results obtained in this study, it may be concluded that the lack of corticosteroid use is the most common risk factor for IVH, and short-term complications may be seen in one-tenth of the cases.

## Introduction

Despite numerous advances in neonatal intensive care, intraventricular hemorrhage (IVH) is a major cause of brain damage, morbidity, and mortality in preterm infants.^1^^,^^2^ However, sometimes, it is also seen in term infants due to thalamic hemorrhage.^3^ Severe asphyxia is recognized as one of the risk factors of IVH in term newborns.^4^ The risk factors of IVH are prematurity, particularly <32 weeks, low birth weight (BW), respiratory distress syndrome, hypoxia, and sepsis.^5^ Very low birth weight (VLBW) infants are at a higher risk of IVH grade 4.^6^ IVH occurs in preterm infants in the germinal matrix after impaired cerebral blood flow and primary hemorrhage.^1^^,^^7^ There are 4 grades for IVH. These measurements are done with ultrasound, based on the amount of blood accumulated in the germinal matrix and lateral ventricles^7^^–^^9^: grade 1 hemorrhage in germinal matrix; grade 2 IVH with 10%-50% ventricle filling without dilatation; grade 3 IVH with more than 50% ventricle filling and with dilatation; and grade 4 hemorrhage in parts of the brain parenchyma on both sides.^10^ Cranial ultrasound is the procedure of choice in the diagnosis of germinal matrix IVH and hydrocephalus.^11^

IVH occurs within the first 5 days after birth: 50% occur on the first day, 25% on the second day, 15% on the third day, and 10% on the fourth day and thereafter. As IVH is identified within 3 days after delivery, it is attributed to improper labor accidents. IVH may also occur before delivery.^12^ Prenatal factors of IVH include chorioamnionitis, mother hemorrhage disorders, respiratory distress syndrome, and prothrombin mutation such as factor V Leiden. Factors such as preeclampsia, delayed clamping of the cord in cesarean section (C/S), and lack of prenatal glucocorticoid therapy are known as protective factors for IVH.^12^^-^^14^ It is now accepted that corticosteroid administration before preterm delivery reduces neonatal mortality and morbidity. However, corticosteroid use in the setting of rupture of membranes remains controversial.^15^ IVH complications include hydrocephaly, depending on the severity of IVH grades 3 and 4. Developmental delay and leukomalacia may occur in the form of cerebral palsy, convulsion, and other neurological disorders.^16^ Approximately 35% of VLBW infants with IVH develop posthemorrhagic hydrocephalus, and 15% of VLBW infants with IVH require intervention.^7^^,^^17^^,^^18^ Not all ventricular dilation progresses to established hydrocephalus that requires treatment; hence, the terms have slightly different meanings.^19^ In this study, we detected hydrocephalus that requires treatment. Neurodevelopmental outcomes of infants with grade 1 and 2 IVH are neurosensory impairment, developmental delay, cerebral palsy, and deafness at 2 to 3 corrected age.^20^ Prognosis after hemorrhage depends on the location and extent of bleeding due to insignificant neurological disorders caused by subarachnoid and subcranial hemorrhage.^16^

However, IVH may cause serious permanent injuries.^21^ Management of IVH and strategies for prevention of brain injury and hydrocephalus are the areas that require further study.^22^ To date, studies have mainly focused on antenatal, prenatal, or postnatal causes of IVH in premature infants separately, and there has been scant research conducted on risk factors for high grades of IVH. We have therefore conducted a control study to deal with short-term complications for these factors and to detect potential risk factors associated with the development of IVH. These data should guide clinicians toward paying adequate attention to those neonates most likely to suffer IVH.

## Materials & Methods

In this retrospective case-control study, 10 000 preterm neonates who were admitted in training hospitals of Alborz University in Karaj from 2014 to 2017 were enrolled through common sampling by ultrasound. This study was approved by the local ethics committee (Abzums.Rec.1395.47). Inclusion criteria were BW < 1500 g, preterm newborns <34 weeks, possibility of follow-up, having no congenital anomaly, and having IVH grade 3 or 4. 

Exclusion criteria were term infants with normal weight, impossibility of being followed up, and the ones having congenital anomaly and IVH grade 1 or 2. Subjects who fulfilled the inclusion criteria were enrolled in the study. The infants with IVH grades 3 and 4 were allocated to the case group. The control group consisted of infants with IVH grades 1 and 2. Their risk factors and short-term complications were assessed. 

Cranial ultrasounds, according to the clinical protocol of the neonatal intensive care unit (NICU), were performed during the third and seventh days and also at the end of the fourth week of life. All of the preterm brain ultrasounds were performed by a neonatal expert radiologist. When there was a clinical suspicion of bleeding, additional ultrasound examinations were performed. Both coronal and sagittal sections were obtained through the anterior fontanels. IVH was classified into 4 grades as described by Papile et al.^23^ Risk factors and short-term complications of IVH were evaluated. A random criterion was considered, based on the assessment of the relation between risk factors and IVH grade. Data were collected in the field. Data collection tools include documentation and data checklists. Data were analyzed by SPSS 19. Mean (SD) was adopted for quantitative variables, and relative and absolute frequency were adopted for qualitative variables. Statistical tests included ANOVA, chi-square, and Fisher’s exact test. The significance level was considered as ≤.05.

## Results

Out of 10 000 neonates, 1203 cases had IVH, of which 767 (63.75%) with grade 1 and 2 IVH were allocated to the control group. A total of 436 (36.25%) consecutive preterm neonates with grade 3 and 4 IVH were allocated to the case group, admitted in Bahonar Hospital, Alborz. 

Out of 436 subjects, 386 (88.6%) cases were small for Gestational age (SGA), and of those 237 (54.3%) cases were male and 196 cases (45.7%) were female. In 2.7% of the subjects, the family history was positive. Delivery methods were C/S and normal vaginal delivery (NVD) in 203 (46.5%) and 287 (65.8%) cases, respectively. Birth rate was the first in 45.7%, second in 40%, third in 10%, and the fourth in 4.3% of the subjects. A total of 231 (52.9%) infants were VLBW and 205 (47.1%) cases were LBW. There was no significant difference in mean maternal age, gestational age (GA), and BW in case and control groups. Infants who were put on mechanical ventilation had 18 times higher odds of IVH (odds ratio [OR] 18.6; 95% confidence interval [CI] 5.09-109.5; *P* < .001). Apgar scores after 1 and 5 minutes were observed, 4.0 ± 2.1 vs. 5.0 ± 1.9 minutes (*P* = .001) and 5.0 ± 1.8 vs. 6.0 ± 1.4 minutes (*P* = .001), respectively, showing their association with use of surfactant in 5.7% of the cases. Risk factors of IVH in our study include chorioammnionitis, low Apgar score (≤5), use of surfactant, mechanical ventilation, pneumothorax, preterm rupture of membrane (PROM), sepsis, use of magnesium sulfate, bleeding, and preeclampsia (Table 1). IVH grade 3 and IVH grade 4 were seen in 311 (71.4%) and 125 (28.6%) cases, respectively. In total, complications were observed in 31 (7.1%) cases including 18 hydrocephalus and 13 mortality. The most frequent risk factor of high-grade IVH was lack of prenatal glucocorticoids in 293 (67.1%) cases. There was 1 maternal mortality. There was no significant statistical difference between infants’ weight and IVH grade (*P* > .05). There was a significant statistical difference between male gender and grade 4 IVH (*P* = .006). There was no significant difference in the frequency of IVH grade, based on complications of IVH in infants (*P* > .05 and based on delivery method (*P* > .05) .There was no significant difference in the frequency of IVH grade, based on birth rate (*P* > .05). Upper IVH grade showed a more significant statistical difference compared to lower GA (*P* = .0001).

**Table 1:** The incidence of risk factors of IVH in the subjects

**Valid**	**Frequency**	**Percent**
Lack of prenatal corticosteroid use Low Apgar (score ≤5) Surfactant Mechanical ventilation Chorioamnionitis Pneumothorax PROM Sepsis Magnesium sulfate Bleeding Preeclampsia Infertility treatment	293 43 25 18 13 11 10 9 5 4 3 2	67.2 10.0 5.7 4.1 2.97 2.5 2.3 2.06 1.14 0.91 0.67 0.45
**Total **	436	100

## Discussion

Our results show the most common risk factors leading to grade 3 and 4 IVH, including lack of maternal corticosteroid use in 293 cases (67.1%), low Apgar score in 43 cases (10%), and use of surfactant in 25 cases (5.7%). In total, complications were observed in 7% of the cases (18 hydrocephalus and 13 mortalities). Our findings, in agreement with the growing number of studies from different populations, report similar concerns about the more immature infants being at a greater risk of severe IVH.

Chevallier et al showed that after adjustment for antenatal magnesium sulfate therapy, level of care in the maternity unit, antenatal corticosteroids, and chest compressions, infants born after placental abruption had a higher risk of grade 3 and 4 IVH.^24^ In our study, the most common risk factor for IVH was lack of use of antenatal corticosteroids.

In a 2010 Korean study, it was suggested that the most important risk factor for IVH is BW lower than 1000 g and that 99 out of 290 infants were under 1000 g. It was also suggested that the incidence of IVH and severity in VLBW infants decrease gradually as GA or BW increases, and that the infants, born after 29 gestational weeks or weighing >1250 g at birth, are at a low risk of IVH.^25^ In a meta-regression and subgroup analysis, Villamor-Martinez et al could not demonstrate an association between the lower GA and BW and the risk of IVH.^26^ In our study, VLBW (in 231 out of 436 cases) was also found to be a risk factor for IVH.

Davis et al, in a retrospective cohort study in 2014 in the United States, showed that risk of mortality and morbidity of infants with IVH grade 4 together with bilateral parenchymal hemorrhage was higher than other grades.^27^ However, in our study, there was no significant difference in complications, based on IVH grades, maybe because of the small size of our study.

Szpecht et al in a retrospective analysis of 35 939 full-term neonates with IVH, reported 2 full-term babies suffering from grade 3 and 4 IVH. They performed cranial ultrasounds and reported that incidence rate of grade 3 and 4 IVH was 5.5 per 100 000 live term births.^28^. However, we studied preterm infants only.

Sajjadian et al, in a study with serial brain sonography in 2009, showed that the incidence of IVH was higher in comparison with other studies. Out of 57 infants with IVH who were enrolled in the study, 40% had grade 1, 11% had grade 2, 25.7% had grade 3, and 2.8% had grade 4 germinal matrix IVH. Hydrocephalus was detected in 20% of the patients who had germinal matrix IVH.^29^ However, they could not detect the complications of IVH.

Merhar et al, in a cohort study in 2012, observed that there were more neurodevelopmental complications in infants with IVH grade 4 compared to other grades.^30^ However, we assessed short-time complications only.

Towers et al, in a study on the effect of transport on the rate of severe IVH in VBLW infants, found that there were 37 cases (11%) of grade 3 or 4 IVH. This study investigated the significant role of transport in IVH complications and grades.^31^

Stoll et al, in a study conducted by NICHD on 9575 infants with GAs between 22 and 28 weeks and BW between 401 and 1500 g, showed that the overall incidence of IVH was 36% in all grades of IVH and increased with decrease in GA. The prevalence of severe IVH also increased with decreasing gestation with rates of 38, 36, 26, 21, 14, and 11 weeks to 7% of the survivors among infants with GA of 22, 23, 24, 25, 26, and 28 weeks, respectively.^32^

O'Leary et al showed that prematurity is the most important neonatal risk factor for IVH too because of preterm infants' germinal matrix fragility and their inability to auto regulate cerebral Blood Flow (CBF).^33^ In our study, lower GA had a significant statistical difference with upper IVH grade, so that the more immature the infant is, the greater the risk of severe IVH is. 

Wei et al determined the association between antenatal steroid administration and IVH rates. They collected cross-sectional data from the California Perinatal Quality Care Collaborative Center during the period 2007 to 2013 from 25 979 VLBW infants with ≤32 weeks of GA. Using multivariable logistic regression, they evaluated the effect of antenatal steroids on IVH, stratified by GA. They showed that antenatal steroid use was associated with a reduction in incidence of any grade of IVH and a reduction in incidence of severe IVH. This association was seen across GAs ranging from 22 to 29 weeks. Also, current guidelines recommend coverage for preterm birth at 24 to 34 weeks of gestation; their results suggest that treatment with antenatal steroids may be beneficial even before 24 weeks of GA.^30^^,^^34^ Also, in our study, lack of prenatal corticosteroid use in 293 cases (67.1%) was the main reason for high-grade IVH. 

In addition, studies of IVH grading have generally shown good results for higher grades of IVH or the absence of IVH, but less so for grade 1 and 2 IVH.^35^^-^^37^. These results are not in line with our results.

Sarkar et al investigated 59 VLBW infants with IVH and reported that 27 cases had IVH grade 3 and 31 cases had IVH grade 4. Infants with IVH grade 4 had a lower BW and GA in comparison to those with IVH grade 3, and also involved more use of MgSO_4._^38^ We assessed short-term complications only. All other risk factors were the same except BW and GA. In addition, short-term morbidities were the same in the 2 groups. We obtained similar results.

One of the limitations of this study was the small sample size and IVH was based on cranial ultrasound rather than magnetic resonance imaging with the possibility of missing mild cases. The retrospective case-control design and the exclusion of a large number of newborns with incomplete data were other limitations of the study. Also, we observed short-term complications in our cases. Therefore, future studies using magnetic resonance imaging to diagnose IVH with a larger sample size, as well as a prospective, properly matched, well-designed cohort format, are needed to confirm our findings. We recommend studies with greater sample sizes and more widespread geographically. 

## Conclusion

 IVH is a multifactorial complication that is more common in preterm and clinically unstable infants. Based on the results of this study and its comparison with other studies in this field, it can be assumed that the most common risk factor for IVH is lack of antenatal corticosteroid use. We recommend the use of corticosteroid for preterm labors to reduce the complications of IVH. We are cognizant of the fact that the results of the study cannot be generalized to the entire population of newborns in Iran. However, the study does present sufficient information to make decision makers more aware of the factors that contribute to mortality in the NICU. 
